# Efficacy of Erbium and CO_2_ Genital Laser Treatment on Genitourinary Syndrome in Female Patients After Breast Cancer: A Scoping Review

**DOI:** 10.1002/lsm.70027

**Published:** 2025-05-14

**Authors:** Keila S. T. Ferreira, Nathalia S. Guimarães, Gisele V. de Oliveira

**Affiliations:** ^1^ Post Graduation Department Faculty of Medical Sciences of MG Belo Horizonte Brazil; ^2^ Department of Nutrition, Nursing School Federal University of MG Belo Horizonte Brazil; ^3^ Santa Casa de Misericórdia de Belo Horizonte Belo Horizonte Brazil

**Keywords:** atrophic vaginitis, cancer survivors, erbium, laser therapy, women

## Abstract

**Objectives:**

This scoping review aimed to synthesize the existing scientific literature on the methods, tools, and strategies employed in vaginal laser therapy for treating genitourinary syndrome in breast cancer survivors.

**Methods:**

A systematic search was conducted in August 2024 across six electronic databases—MEDLINE (via PubMed), Embase (Elsevier), Scopus (Elsevier), Cochrane Library (Central), Virtual Health Library (VHL), and Web of Science (Clarivate Analytics)—for studies reporting the use of CO_2_ or erbium lasers in breast cancer survivors. The search included articles in all languages and used MeSH terms and database‐specific adaptations.

**Results:**

A total of 2372 studies were identified through the electronic databases. After excluding 458 duplicates, 2134 titles and abstracts were screened. Thirty full‐text records were assessed for eligibility, of which 10 were excluded for incomplete results and different technologies. Ultimately, 1189 patients out of 20 studies were selected for inclusion in this review: 14 studies used CO_2_ laser, 5 used erbium laser, and 1 used both technologies. This review showed that use of vaginal lasers to approach GSM in BSC patients is being used since 2017, and 13 out of 20 studies concluded for the safety and efficacy of vaginal laser to treat GSM in BCS patients, comparable to standard treatment.

**Conclusion:**

Both CO_2_ and erbium lasers appear to be safe and effective treatment options for breast cancer survivors with genitourinary syndrome. New larger, multicenter studies are needed to enhance safety, standardize treatment protocols, and provide further evidence on the efficacy of these therapies in this patient population.

## 1 Introduction

Breast cancer is the most frequently diagnosed cancer in women worldwide [1]. Around 42%–70% of patients undergoing systemic treatment for breast cancer, including endocrine therapy, are likely to experience some degree of vulvovaginal atrophy (VVA) [2]. Chemotherapy can cause sudden temporary or permanent menopause in up to 80% of premenopausal women due to premature ovarian failure, and it can also lead to further reductions in estrogen levels after natural menopause [3]. The climacteric phase can change the epithelial lining of the genital tract, due to decreasing estrogen levels, further leading to the onset of symptoms and signs characteristic of genitourinary syndrome of menopause (GSM) [4]. The initial symptoms of VVA include reduced vaginal lubrication, followed by additional vaginal and urinary symptoms that can worsen with secondary infections, such as burning, itching, bleeding, discharge, pain during intercourse, and painful urination [5]. Nonhormonal options, such as lubricants, are not effective enough in fully alleviating atrophy symptoms. While local estrogens are the standard treatment for GSM, they may adversely affect breast cancer outcomes and therefore they are usually avoided in this patient group [6].

Recent studies indicate that laser therapy could serve as a beneficial nonhormonal treatment option to address postmenopausal genitourinary syndrome, as well as for a group of women who survived breast cancer, and could not use hormones to treat the syndrome [7]. A preliminary search of PROSPERO, MEDLINE, the Cochrane Database of Systematic Reviews, and JBI Evidence Synthesis were conducted, and no current or progress scoping reviews on the topic were identified. Mension et al., a study in Scopus (Elsevier) has compared several therapies used to treat genitourinary syndrome on breast cancer survivors (BCS) [8]. The present study, however, focuses on Laser Therapy using either Erbium or CO_2_, to address the genitourinary syndrome on patients with breast cancer, including several studies that have been published after the publication of Mension et al. [8]. This review aims to provide data for physicians and other health professionals who deal directly with BCS, so that they have access to more information about a possible safe and effective alternative for the treatment of GSM. In this context, we structured this review to answer two questions: “Are CO_2_ and erbium lasers safe treatments for BCS?” “Is laser treatment effective to treat genitourinary syndrome in breast cancer patients?”

## 2 Materials and Methods

This study was written as a scoping review according to JBI tools, registered on the Open Science Framework platform (https://osf.io/9xuys/, accessed on June 28, 2024).

### 2.1 Data Source and Search Strategy

**Figure 1:**
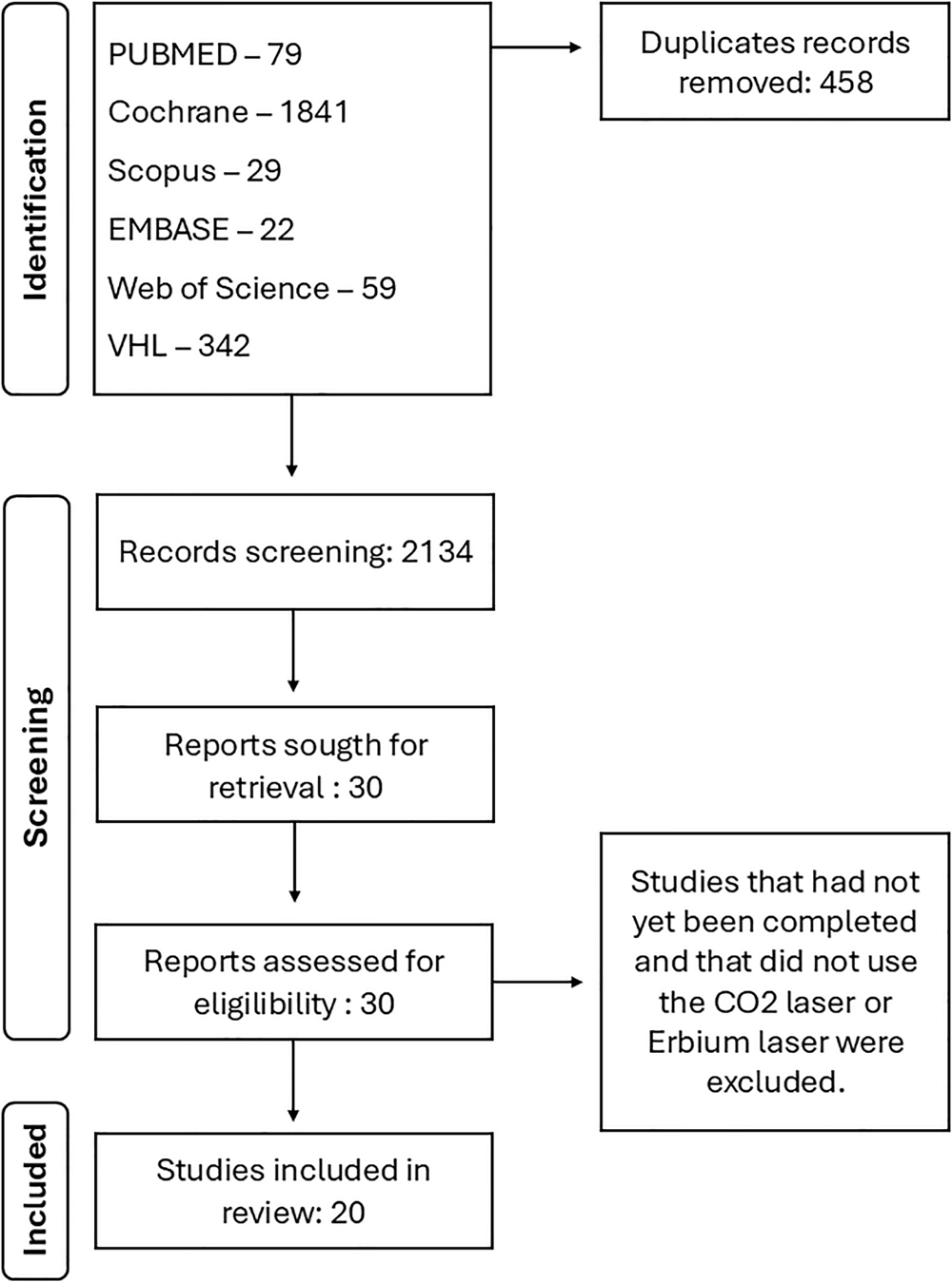
Studies included in this review. A total of 2372 studies were identified through the electronic databases. After excluding 458 duplicates, 2134 titles and abstracts were screened. Thirty full‐text records were assessed for eligibility, of which nine were excluded for incomplete results and one for using a non‐ablative 1470 nm laser. Ultimately, 20 studies were selected for inclusion in this review: 14 using CO_2_ laser, 5 using erbium laser, and 1 comparing both technologies.

Preliminary search of MEDLINE (by PubMed) and CENTRAL (by Cochrane Library) were performed to identify articles on the topic. Text words contained in the titles and abstracts of relevant articles, as well as index terms used to describe the articles, were used to develop a comprehensive search strategy for MEDLINE (via PubMed), Embase (Elsevier), Scopus (Elsevier), CENTRAL (via Cochrane Library), and the Virtual Health Library electronic databases. Western Pacific Region Index Medicus OR Spanish Bibliographic Index in Health Sciences OR Latin American and Caribbean Literature in Health Sciences OR Peruvian Literature in Health Sciences OR National Bibliography in Health Sciences OR Health Sciences Descriptors OR Research Guidance Service, and Web of Science (by Clarivate Analytics). The search strategy, including all identified keywords and index terms, was adapted for each included information source (Table 1). The reference lists of all selected studies underwent critical appraisal and were screened for additional studies (Figure 1). There was no language limitation for the search. Unpublished study sources/gray literature were not searched or included in this review. Although the date of the studies was not limited, the oldest study was from 2017.

**Table 1:** The study search strategy, including all identified keywords and index terms.

Search	Query
1	(title:((title:(Cancer Survivors) OR abstract:(Cancer Survivors)) OR (title:(Atrophic Vaginitis) OR abstract:(Atrophic Vaginitis)) AND (title:(Laser Therapy) OR abstract:(Laser Therapy)) OR (title:(Erbium) OR abstract:(Erbium)) AND (title:(Lasers, Gas) OR abstract:(Lasers, Gas))) OR abstract:((title:(Cancer Survivors) OR abstract:(Cancer Survivors)) OR (title:(Atrophic Vaginitis) OR abstract:(Atrophic Vaginitis)) AND (title:(Laser Therapy) OR abstract:(Laser Therapy)) OR (title:(Erbium) OR abstract:(Erbium)) AND (title:(Lasers, Gas) OR abstract:(Lasers, Gas))))
2	(((Cancer Survivors [Title/Abstract])) OR (Atrophic Vaginitis [Title/Abstract])AND ((Laser Therapy) OR (Erbium[Title/Abstract]) OR (Lasers, Gas[Title/Abstract])))
3	Women AND Cancer Survivors in Title Abstract Keyword OR Atrophic Vaginitis in Title Abstract Keyword AND (Laser Therapy) OR (Erbium) OR (Lasers, Gas)
4	(TITLE‐ABS‐KEY (women) OR TITLE‐ABS‐KEY (“Cancer Survivors”) AND TITLE‐ABS‐KEY (atrophic AND vaginitis) AND TITLE‐ABS‐KEY (laser AND therapy) OR TITLE‐ABS‐KEY (erbium) OR TITLE‐ABS‐KEY (“Lasers, Gas”))
5	((‘cancer survivor’/exp OR ‘cancer survivor’) AND ‘atrophic vaginitis’:ti,ab,kw AND ‘laser therapy’:ti,ab,kw OR erbium:ti,ab,kw OR ‘gas laser’:ti,ab,kw) AND female:ti,ab,kw AND ([controlled clinical trial]/lim OR [randomized controlled trial]/lim)
6	(((((TI=(Cancer Survivors)) AND TI=(Atrophic Vaginitis)) AND TI=(Laser Therapy)) OR TI=(Erbium)) OR TI=(Lasers, Gas)) AND ALL=(female)
7	(cancer survivors) OR (atrophic vaginitis) AND (laser therapy) OR (erbium) OR (lasers, gas) AND (db:(“MEDLINE” OR “WPRIM” OR “IBECS” OR “LILACS” OR “LIPECS” OR “BINACIS” OR “DECS” OR “SOF”))
8	Cancer Survivors; Atrophic Vaginitis; Laser Therapy; Erbium; Lasers, Gas
No limits of date or language.	

*Note:* A preliminary search of MEDLINE and Cochrane were performed; text words contained in the titles and abstracts and index terms used to describe the articles were used to develop a comprehensive search strategy for MEDLINE (by PubMed), Embase (Elsevier), Scopus (Elsevier), CENTRAL (by Cochrane Library), “WPRIM,” “IBECS,” “LILACS,” “LIPECS,” “BINACIS,” “DECS,” “SOF” by Virtual Health Library and Web of Science (Clarivate Analytics).

### 2.2 Eligibility Criteria

Clinical studies that evaluated the action of CO_2_ laser and erbium laser for the treatment of GSM in BCS were included.

Studies that did not evaluate the action of lasers involved patients who were not BCS, or included survivors of other types of cancers were excluded. Other literature reviews were also excluded.

### 2.3 Study Selection and Data Extraction

The results of the electronic searches conducted in the defined databases were uploaded to the Rayyan app, from the Qatar Computing Research Institute, for systematic reviews. Two reviewers independently analyzed the titles and abstracts. These reviewers also independently assessed each eligible study to determine whether they met the inclusion criteria. A third independent reviewer resolved any discrepancies.

The research team prepared and applied a data extraction spreadsheet to summarize the following information from the studies: reference (name and year of publication), study location (country), title, objectives, follow‐up period, type of laser used, assessment methods (Table 2), main results, and conclusions. All reports on adverse effects and complications during and after the procedure were extracted from the studies and summarized in Table 3.

**Table 2:** Assessment methods used in the study.

Method	Number of studies	Studies
VHI (Vaginal Health Index)	13	1, 2, 5, 6, 7, 11, 12, 13, 15, 16, 19, 20, 21
FSFI (Female Sexual Function Index)	10	3, 7, 10, 12, 13, 14, 15, 17, 19, 20
VAS (Vaginal Assessment Scale)	10	3, 4, 5, 9, 13, 15, 16, 17, 19, 20
Intensity of symptoms	3	7, 9, 17
Biopsy	4	4, 12, 20, 21
Lickert scale	3	4, 5, 7
Microbiota analysis	2	7, 2
Female sexual distressful scale revised	3	10, 13, 14
EOLTC‐QLQ BR45 (European Organization for Research and Treatment of Cancer‐Quality of Life Questionnaire in Patients with Breast Cancer)	3	11, 12, 13
UDI‐6 (urinary symptoms)	1	20
Vaginal elasticity	1	12
Sexual frequency	1	17
Urinary stress	1	3
Urinary function	1	10
Vaginal citokine analysis	1	7
POP symptoms (Pelvic Organ Prolapse Questionaire)	1	11
VST (vulvodynea swab test)	1	15

*Note:* Table presents the assessment methods and the respective studies that utilized them. The most frequently used methods were VHI and FSFI, utilized by 13 and 10 studies, respectively.

**Table 3:** Adverse effects reported for breast cancer survivor patients treated with CO_2_ and erbium vaginal lasers.

Authors	Study	Number of patients	Laser	Number of sessions	Adverse events during the procedure	Adverse events after the procedure
Sikkiquini et al. [1]	Retrospective observational study	45	CO_2_	3	Mild discomfort	Not reported
Hersant et al. [2]	Prospective clinical study	20	CO_2_	2	Mild pain, moderate bleeding (patients)	Not reported
Quick et al. [3]	Prospective	64	CO_2_	3	Mild discomfort	Transient vaginal discharge grade 1–2 (54.3%), transient vaginal dryness (23.6%)
Fernandes et al. [4]	Randomized, controlled, multicenter clinical trial	70	CO_2_	3	Mild pain	Not reported
Pieralli et al. [5]	Prospective, descriptive, uncontrolled cohort study	50	CO_2_	3	Slight pain when inserting the probe (24%)	Not reported
Monthes et al. [6]	Retrospective observational clinical study	16	Erbium Yag	1	Not reported	Not reported
Becorpi et al. [7]	Prospective clinical study	20	CO_2_	2	Not reported	Not reported
Pagano et al. [9]	Retrospective observational clinical study	82	CO_2_	3	Pain during insertion of the probe (95%) pain during movement of the probe (94%) pain associated with the laser (87%)	Not reported
Quick et al. [10]	Prospective clinical study	64	CO_2_	3	Not reported	Not reported
Gold et al. [11]	Randomized controlled clinical trial	43	Erbium Yag	2	Not reported	Not reported
Mension et al. [12]	Randomized, double‐blind, placebo‐controlled clinical trial	72	CO_2_	5	Spotting (light bleeding), vaginal itching, mild pain during application	Moderate complications, such as urinary tract infections (10%)
Lami et al. [13]	Prospective clinical study	26	CO_2_	3	Mild discomfort	Not reported
Quick et al. [14]	Prospective clinical study	39	CO_2_	3	Not reported	Not reported
Ocui et al. [15]	Retrospective clinical study	204	Erbium Yag	3	Mild pain and heat	Discharge, itching, dryness, swelling, incontinence (transient)
Gambacciani et al. [16]	Pilot, prospective and longitudinal clinical study	37	Erbium Yag	3	Not reported	Not reported
Flint et al. [17]	Multicenter, randomized, controlled clinical trial	88	CO_2_ x Erbium Yag	3	Not reported	Not reported
Fidecicchi et al. [18]	Pilot, prospective, randomized, comparative, double‐blind clinical study	68	Erbium Yag	3	Transitory warm	Self‐limiting leukorrhea
Pearson et al. [19]	Prospective pilot clinical study	45	CO_2_	3	Not reported	Not reported
Jacobsen et al. [20]	Prospective, randomized, comparative, double‐blind clinical study	90	CO_2_	5	Not reported	Not reported
Lee et al. [21]	Prospective, randomized, comparative, double‐blind clinical study	46	CO_2_	3	Not reported	Not reported
Total	20	1189	15 CO_2_ and 6 ERBIUM	1–5	Four reported mild discomfort, two reported heat and transitory warm, pain was reported during the application in five studies and bleeding in two studies	Sixteen studies have not reported any adverse effects after the procedure, two reported vaginal discharge, one reported limited leukorrhea, two reported itching, two studies reported dryness, swelling was reported by one patient, transient incontinence was reported by one study

*Note:* This review retrieved 20 studies that used 1–5 sessions of vaginal lasers to treat BCS patients with genitourinary syndrome, 14 out of 20 have used CO_2_ laser, four used Erbium laser, and one reported using both technologies, including 1189 patients. Immediate side effects included Mild discomfort, reported in two studies, heat and transitory warm in two studies, pain during the procedure, in five studies, and bleeding, in two studies. Complications after the procedure were not reported in 16 out of 20 studies, while two reported vaginal discharge, one reported limited leukorrhea, two mention itching, two studies reported dryness, swelling was seen in one study, as well as transient incontinence.

## 3 Results

A total of 2372 studies were found through electronic databases and added to Rayyan. Using the software, 458 duplicates were excluded, and after that, 2134 titles and abstracts were examined. Of these, 30 articles were fully assessed and evaluated according to the eligibility criteria; nine were excluded for not presenting complete results, and one for using a different wavelength, a nonablative 1470 laser. After this evaluation, 20 studies were selected for this review, 14 using CO_2_ laser, five using Erbium laser, and one comparing CO_2_ and Erbium (Figure 1). Nineteen studies were conducted exclusively with BCS. One of them involved a total of 135 women, 45 of whom were BCS [1]. Only studies with BCS were included in the review (Figure 1).

The total number of patients in the studies was 1189. Among the analyzed studies, 14 used the CO_2_ laser [1, 2, 3, 4, 5, 7, 9, 10, 12, 13, 14, 19, 20, 21], five used the erbium laser [6, 11, 15, 16, 18], and one study compared the erbium laser with the CO_2_ laser [17]. Of these, 10 studies did not include a control group [1, 2, 3, 5, 6, 7, 9, 10, 13, 16]. Among those with a control group, five used sham treatment [12, 14, 19, 20, 21], two used a different protocol or laser type [15, 18], one used promestriene [4], one used estriol [19], one used intravaginal hyaluronic acid [11], and one compared the laser to standard nonhormonal therapies, such as moisturizers, lubricants, and vibrators, combined with sham treatment [12]. Immediate side effects included mild discomfort, reported in two studies, heat and transitory warm in two studies, pain during the procedure, in five studies, and bleeding, in two studies. Complications after the procedure were not reported in 16 out of 20 studies, while two reported vaginal discharge, one reported limited leukorrhea, two mention itching, two studies reported dryness, swelling was seen in one study, as well as transient incontinence. Urinary tract infection was reported by Mension et al., occurring in 10% of the 72 patients (Table 3).

Fourteen studies performed three laser sessions [1, 3, 4, 5, 9, 10, 13, 15, 16, 18, 19, 21], three studies performed two sessions [2, 7, 11], two studies performed five sessions [12, 20], and one study performed only one session [6], resulting in an average of 2.95 sessions per study. The patients were followed for an average of 8.6 months, with 1 month being the shortest follow‐up time and 24 months the longest.

For assessment methods, 13 studies used the Vaginal Health Index (VHI), 10 studies used the Female Sexual Function Index (FSFI), 10 studies used a visual analog scale (VAS), three studies used a Likert scale, and four studies performed biopsies [4, 12, 20, 21]. One of the biopsy studies also included Masson's Trichrome staining and immunohistochemistry [20]. Other evaluation tools are detailed in Table 2.

Of the 20 studies, 13 concluded that both CO_2_ and erbium lasers are safe and effective alternatives for the treatment of GSM, especially for BCS who have limited treatment options due to the inability to use hormonal therapy [1, 2, 3, 5, 6, 7, 9, 10, 13, 14, 16, 19, 20]. Three studies concluded that the improvement achieved with the laser is comparable to that achieved with standard treatments [4, 11, 12]. One study concluded that the improvement provided by the laser is similar to the improvement achieved with standard treatment, but with greater discomfort compared to topical treatment [12]. Another study concludes that there is no significant improvement [21].

## 4 Discussion

GSM was a term coined by the International Society for the Study of Women's Sexual Health and the North American Menopause Society. It refers to a number of symptoms due to loss of estrogen that leads to atrophy in the vulvovaginal area, leading to sexual dysfunction, vaginitis, bladder‐urethral symptoms, recurrent urinary tract infections and postcoital bleeding, among others [22]. While it is efficiently managed with the use of hormone‐therapy, the syndrome is particularly severe in the group of breast cancer survivor patients (BCSs), in which the ovarian function can be either affected by chemotherapy and anti‐estrogenic treatments.

Data shows that BCSs experience continued difficulty with vaginal health and sexual activity for 5 or more years after breast cancer treatment. The treatment of genitourinary Syndrome in BCSs is aggravated by three main factors: women may experience difficulties and seeking for help regarding this subject; clinicians are uncertain about symptoms treatments, and there is currently a lack of therapeutic options for this group of patients [22].

In this scenario, laser therapy has gained increasing popularity as a possible nonhormonal option treatment to the genitourinary syndrome [7]. However, it is still not employed in large scale to treat BCS, as the fast atrophy of the genital area induced by the cancer treatment frequently leads to acute symptoms that include easy vaginal laceration and bleeding and most clinicians are uncertain whether laser treatment would be safe and effective to treat this group.

Recently, Jung et al. have shown in a systematic review and meta‐analysis that vaginal laser treatment is associated with similar improvement in genitourinary symptoms as vaginal estrogen therapy, pointing that further research would be needed to test whether vaginal laser therapy could be a potential treatment option for women with contraindications to vaginal estrogen [23]. This scoping review systematically looked for all studies investigating the use of fractional CO_2_ and erbium lasers to treat the genitourinary syndrome in BCSs. We were able to analyze the data of 1189 patients treated with lasers, among 20 studies, that reported only mild and transient side‐effects; urinary tract infection, although reported in only one out of 20 studies, might pose a concern, and deserves investigation in further studies (Table 3). According to the data evaluated in this review, lasers can indeed provide safe and effective treatment for genitourinary syndrome, mainly in BCSs, where treatment options are limited [1, 2, 3, 5, 6, 7, 9, 10, 13, 14, 16, 19, 20]. The improvement of such treatment employing lasers seems to be comparable to standard treatments [4, 11].

## 5 Conclusion

This scoping review gathered data that suggests that fractional erbium and CO_2_ lasers can be considered as safe and effective alternatives to approach genitourinary syndrome in BCS, a group of patients with very limited options of treatment. Vaginal Lasers seem to be comparable to standard treatments for GUS. New larger, multicenter studies should confirm this promising method of treatment for BCS.

## Conflicts of Interest

Gisele V. de Oliveira is a speaker for Vydence and LMG (Laser Industries) but she has not received any compensation for this study. The remaining authors declare no conflicts of interest.
